# A Reliable TTP-Based Infrastructure with Low Sensor Resource Consumption for the Smart Home Multi-Platform

**DOI:** 10.3390/s16071036

**Published:** 2016-07-05

**Authors:** Jungho Kang, Mansik Kim, Jong Hyuk Park

**Affiliations:** 1 School of Computer Science & Engineering, Soongsil University, 369 Sangdo-ro, Dongjak-gu, Seoul 06978, Korea; kjh7548@ssu.ac.kr (J.K.); mansik@ssu.ac.kr (M.K.); 2 Department of Computer Science and Engineering, Seoul National University of Science and Technology (Seoul Tech), 232 Gongneung-ro, Nowon-gu, Seoul 01811, Korea

**Keywords:** smart home, PUFs, TTP, multiplatform, resource

## Abstract

With the ICT technology making great progress in the smart home environment, the ubiquitous environment is rapidly emerging all over the world, but problems are also increasing proportionally to the rapid growth of the smart home market such as multiplatform heterogeneity and new security threats. In addition, the smart home sensors have so low computing resources that they cannot process complicated computation tasks, which is required to create a proper security environment. A service provider also faces overhead in processing data from a rapidly increasing number of sensors. This paper aimed to propose a scheme to build infrastructure in which communication entities can securely authenticate and design security channel with physically unclonable PUFs and the TTP that smart home communication entities can rely on. In addition, we analyze and evaluate the proposed scheme for security and performance and prove that it can build secure channels with low resources. Finally, we expect that the proposed scheme can be helpful for secure communication with low resources in future smart home multiplatforms.

## 1. Introduction

Thanks to advancing ICT technology, we live in ubiquitous environment where people can get IT services anytime and anywhere. IoT is one of the representative technologies for the ubiquitous environment. By technology, many things are organically connected to the web to provide various services to the users [1,2,3]. It is applied to a wide range of areas including smart agriculture, smart homes, and connected cars [4,5]. Among the application areas, the smart home market has been growing exponentially all over the world as IoT smart devices develop and the services and R&D areas using smart devices expand [6]. Strategy Analytics reported that the world smart home market has grown by 10% annually and the market size will reach 115 billion US dollars by 2019 [7]. Harbor Research also forecasted that the number of IoT terminals installed around the world will be 8 billion units by 2020 and 47% of them will be installed in smart homes. In addition, Gartner expects that the number of smart home-related devices will reach 6.96 billion units by 2020. The world market demand for a new growth engine encourages many companies to participate in the smart home market. According to ADL, a strategic consulting company, the participants in the smart home market can be divided to: (1) electrical power distribution/building automation players; (2) smart building control specialists, (3) building application players; (4) household appliance players; (5) SW, IT, communication equipment players and (6) service providers [8]. Because smart home services are based on fusion technologies and provided on multiple platforms, various heterogeneities exist in the smart home service environment. Furthermore, various kinds of security threats are posed against user’s privacy and service reliability as the smart home market grows. In February 2015, HP warned in its research report that most of the smart home IoT devices are vulnerable to security threats regarding password, encryption and authentication procedures, and can be easily exposed to cyber-crime because user has to provide personal information to use smart home IT devices [9]. According to a Symantec report on the security of smart home devices, they have authentication weakness [10]. Moreover, various sensors used for the smart home have low computing resources. They cannot process the complicated computation tasks required to perform mutual authentication and build secure channels. Even a service provider that has enough computing resources can face overheads in processing data from an exponentially increasing number of smart home sensors [11,12,13,14]. To provide secure and flexible services for the multiplatform smart home environment, the infrastructure is required to connect with multiplatforms and to support low resources. This paper proposes an infrastructure based on Trusted Third Party (TTP) by which mutual authentication and communication can be secured in a multiplatform smart home environment and a scheme to design authentication and secure channels with minimum resources, using PUFs to solve the abovementioned problems.

This study is composed as follows: Section 2 will describe the smart home infrastructure, security smart home requirements, and previous research on smart homes. Section 3 will describe the proposed smart home infrastructure and a protocol for mutual authentication and secure channels in details. Section 4 provides an analysis of the computing resources and storage resources of the proposed scheme, while they will be discussed in Section 5.

## 2. Related Works

In this section, we discuss the infrastructure of and security requirements for smart homes, and review previous related works. 

### 2.1. Smart Home Infrastructure

**Figure 1:**
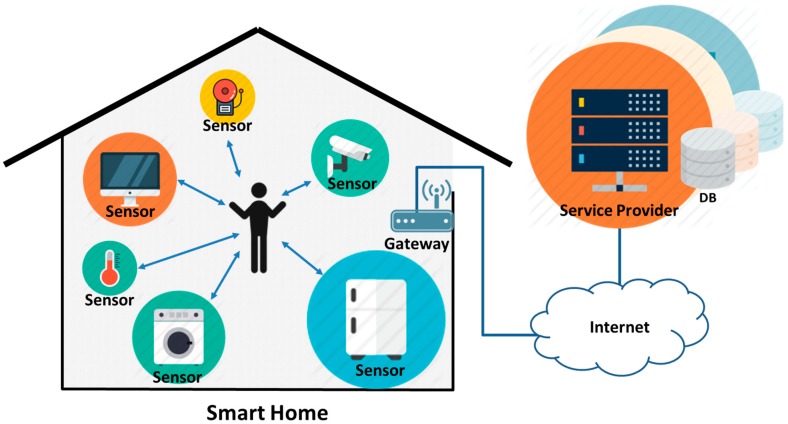
Smart home infrastructure.

In general, the smart home infrastructure consists of sensors and a gateway, and service providers, as seen in Figure 1. In the smart home, those sensors provide a single service directly to home occupants, using information they collect in real time, or multiple services through exchanging information with other sensors [15]. When the sensors transmit collected information to a service provider, they have to go through a gateway to communicate with the outside because they have low resources for use. The gateway functions in two ways: it receives the data the home sensors collect in the smart home and sends the date to service providers or transmits the data from the service providers to the sensors. In addition, the gateway directly sends a control command to the sensors on the basis of the received information and depending on the type of service. Service provider is a server registered for service provision before a sensor is deployed in the smart home. It receives information sensed by a sensor through the gateway and gives a control command or service. Since the smart home infrastructure is mainly built with sensors, gateway and service providers, various and numerous various industries are involved [6], creating a multiplatform environment.

### 2.2. Security Requirements for Smart Home

A variety of security threats exist in smart home environment, just like in existing ICT environments. Therefore, it is necessary to ensure the security and privacy of smart home services. In designing a smart home environment, particular consideration needs to be paid for the multiplatform heterogeneity and limited resource requirements for sensors of low capacity.

*Multiplatform*: In the smart home, various industries are involved such as server, control and communication required inside of the smart home, as well as smart sensor manufacturing. Therefore, the sensors and the service providers exchange information on heterogeneous smart home multiplatforms [16,17,18,19]. Allseen Alliance, OIC, Thread Group, HomeKit, IIC have currently suggested global standards to improve the multiplatform-based smart home environment, but none of them stands out as a market leader yet. Moreover, since there is no preemptive standard in the market although each standard provides the users and developers with their own security tools and managers, there are lots of challenges to connect with other platforms in security aspects. In such a smart home environment, heterogeneous sensors and/or service providers have to organically communicate with each other in a multiplatform environment so as to provide uniformed or common services to home occupants.

*Security*: As IoT technology has grown geometrically, so have security threats grown proportionally. Particularly, the smart home is a complex environment combined with various services. Therefore, it can easily be vulnerable to various security threats and malicious attacks can cause serious damage or even threaten the lives of the occupants in the home environment [20]. Access control by mutual authentication must be in place to protect a user from malicious attacks and ensure secure communication [21]. Furthermore, a security channel has to be established, besides mutual authentication, to protect communications from threats such as relay attacks, replay attacks, leaked keys, and forward security. As a way to build a secure sensor environment, Tien-Dung et al. [22] and Mohammad et al. [23] proposed ELK and LHK, respectively, but they are not suitable for the smart home environment. In addition, Adrian et al.’s CoGKTK [24] and Wong et al.’s sGIM [25], which are based on the group multicast transmission technique, also turned out to be unsecure to various attacks.

*Privacy*: In general, the low capacity smart home sensors exchange information with the service providers through the gateway to provide services. Especially while they are communicating, the personal information of home occupants can leak out due to malicious eavesdropping from outside or inside entities, or user’s carelessness, which can eventually result in the breach of privacy to a great extent [26]. Therefore, it is necessary to build a secure channel for each interrelation of the components in the smart home—between the smart home sensors and the gateway; between the gateway and the service providers; between the smart home sensors and the service providers—to protect the occupants’ privacy from internal/external security threats and minimize unintended information exposure.

*Low Resources*: A variety of sensors are used in the smart home. Some of them have abundant computing resources. Such sensors are used in refrigerators, washing machines, and TVs, while others have limited power or computing resources [27,28]. The latter are mainly used for electric lights, thermometers, alarm clocks, and so on. The low resource sensors use small batteries and low-capacity chips to balance performance and cost [29]. As a result, smart home sensors can neither perform complicated computations nor store much data due to their small memory capacity. Particularly, this resource limitation of a sensor is a great hurdle to creating secure channels to protect a user from malicious attacks or breaches of privacy. This is why a light-weighted method, by which a secure channel can be built only with simple computation and low memory, is required for a smart home sensor. 

### 2.3. Previous Research on Smart Home

In this section, previous studies related to smart home service and security are reviewed. Jinsung et al. [30] proposed a ZigBee-based intelligent self-adjusting sensor (ZiSAS) as a way to improve the operational efficiency of wireless sensor networks (WSNs), which aims to balance the cost and performance of a sensor in the smart home environment. With consideration of the cost and performance constraints of a smart home sensor, the ZigBee (IEEE 802.15.4 standard) ZiSAS provides four functions as follows to provide efficient intelligent service: first, it provides a flexible middleware architecture for the smart home environment. The flexible middleware architecture consists of three layers in which a sensor can provide services dynamically in the smart home environment. Second, it provides situation-based sensor self-adjusting function to relieve the WSN’s hardware/software limitations. Third, it provides event-based sensor control in the smart home environment. Event- based sensor control prevents unnecessary information gathering from all the sensors. Finally, it provides occupants with context-aware services based on sensors’ continuous collection of environmental information and analysis of current situations. In addition, Jinsung et al. also showed prototype smart home services using the proposed system. However, it has security and privacy protection problems because not only general data but also the movement of residents detected in ZiSAS are not encrypted to be exchanged. In addition, it has the problem of not supporting multiplatform use without considering the multiplicity among the heterogeneous sensors.

Alessandro et al. [31] proposed a wireless architecture in which the presence, movement, and behaviors of occupants in a smart home are estimated; the power of the smart home is managed; and elderly people are monitored. The proposed wireless architecture is a flexible wireless architecture aiming to satisfy both user acceptance and system performance demands based on the collection of large-scale data sets and training regarding smart home occupants’ location and behavior. In the flexible wireless architecture, heterogeneous wireless devices also abstract information physically collected from other devices through the abstraction layers of software stacks in the multiplatform environment, so that service developers can get access to physical data without worrying about hardware compatibility and provide services at the upper layer. In addition, they proposed an integrated and low-cost wireless architecture to guarantee two important key points for future smart homes: user acceptance and low system complexity. However, the proposed model is not encrypted in spite of the sensitive information about the movement of the residents measured in the smart home, therefore, it has the possible issue of violating user privacy and it does not use secure channels for the communication among the sensors.

The smart home has several issues: smart home sensors of low power and low computing resources; the heterogeneity of devices that have to communicate in a multiplatform environment; and, as mentioned above, possible breaches of privacy by malicious attacks or unauthorized access from users’ reckless management. To provide secure remote access to home automation networks ‘anywhere and anytime’, Khusvinder et al. [32] proposed a remote access framework based on a Remote Home Server (RHS) provided by a trusted third party. The proposed framework is a hybrid scheme of direct and third party-based remote communication approaches. It overcomes the weaknesses of existing direct access and third party-based access schemes by a trusted third party having a none-home owner search the IP address of the automated home and allowing a remote user that gets an IP address from the RHS have a direct access to the home. In addition, the system enhances the advance security by distributing the master key shared between the home automation system and RHS as well as one between the home automation system and mobile clients, randomly creating session keys every session, and thus building a secure channel. In addition, the researchers proposed a ZigBee-based home gateway through which the home automation system is connected to the external Internet at low cost and with standalone devices.

As smart home appliances such as lights, smoke-alarms, power switches, baby monitors, and so on have increased in number exponentially, Vijay et al. [33] provided network-level protection, as a solution to maintain privacy and provide security from snooping or intrusion into the family’s activities, that system can monitor network activities and detect suspicious behaviors. The proposed scheme applies dynamic security rules with software defined networking (SDN) technology that can block and/or quarantine devices dynamically, based on the home context such as time-of-day or occupancy of a house. Furthermore, they suggested Security Management Provider (SMP) as an external entity that develops, customize, and delivers user extra safeguards for smart home devices at the network level. It is a three-party architecture that consists of SMP role, ISP/home-router-vendor role, and consumer role providing security-as-a-service.

## 3. Proposed Infrastructure

### 3.1. Proposed Smarthome Multiplatform Infrastructure

**Figure 2:**
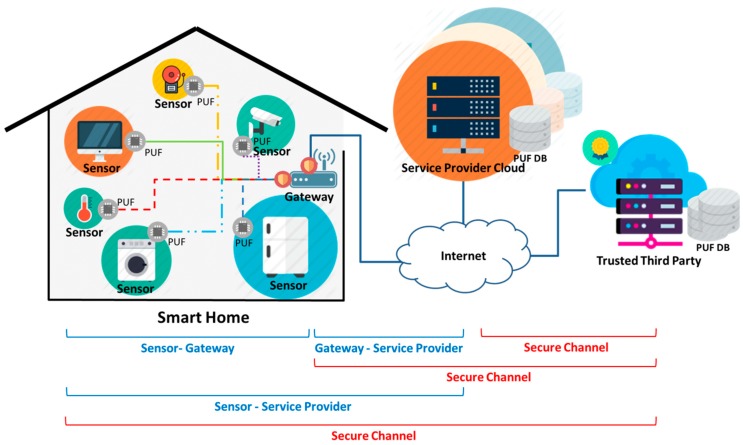
Proposed smart home multiplatform infrastructure.

Figure 2 shows the proposed infrastructure for multiplatform smart home environments. In the smart home, there are various smart home sensor devices, which are registered with different service providers (SPs) due to their different manufacturers or services and work on the multiplatform. Therefore, they cause the issue of heterogeneity. That is why the gateway exists to solve the heterogeneity problem and open a single communication channel to the outside. Outside the smart home, several SPs exist by sensor platform, form clouds, and communicate with the smart home sensors through the gateway. Finally, TTP exists to solve the heterogeneity between various sensors and SPs and secure the reliability of the communication entities. TTP is in a reliable relation with sensors and all the sensors have physically unclonable PUFs which can process challenge-response and key generation with low resources [34,35,36,37]. The proposed multiplatform smart home infrastructure processes, prior to building a secure channel, mutual authentication between the sensors and the gateway, between the gateway and SP(s), and between SP(s) and the sensors, which are involved in communication by entity.

### 3.2. Proposed Protocols

Using TTP in the proposed multiplatform smart home environment, the efficient mutual authentication process consists of two stages: the provisioning stage and the authentication stage. In the provisioning stage, each service provider, smart home sensor devices and a gateway are registered on TTP. In the authentication stage, mutual authentication is carried out between smart home sensor devices and the gateway, between the gateway and service providers, and between smart home sensor devices and service providers and the session key with which a security channel can be established is allocated to smart home sensor devices and service providers. It is assumed that TTP, sensors, and TTP are in and gateway and TTP and SP are reliable. The parameters for the proposed protocol are listed in Table 1.

#### 3.2.1. Provisioning Phase

**Figure 3:**
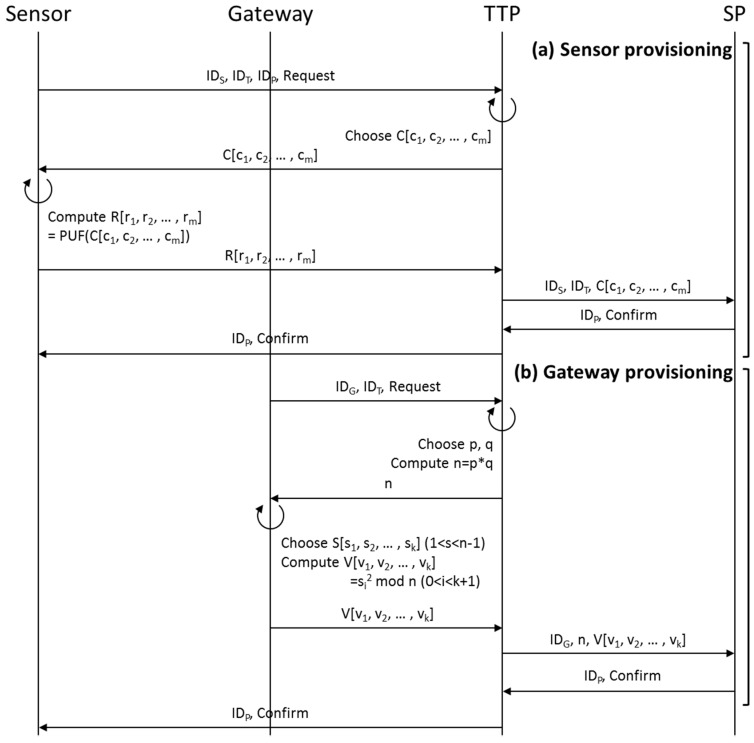
Provisioning phase: (**a**) sensor provisioning; (**b**) gateway provisioning.

Sensors and the gateway are registered on TTP and SP at the provisioning state as shown in Figure 3 prior to being deployed at the field. First, in the (a) sensor provisioning stage (Figure 1), a sensor sends to TTP, through a secure channel, the message asking it (TTP) to register its own ID and ID of TTP and SP that it wants to register. When the TTP is asked to register IDs from a sensor, it randomly selects Challenge vector C[c_1_, c_2_, … , c_m_] and sends it to the sensor to construct the PUF DB of the relevant sensor. The sensor takes the Challenge vector C from TTP as input value to compute the Response vector R[r_1_, r_2_, … , r_m_] through PUF and responds to TTP. Then TTP maps Challenge vector C and Response vector R, constructs the PUF DB for concerned sensor, transmits to the SP (that the sensor requested to register) its own ID, Challenge vector C[c_1_, c_2_, … , c_m_] and ID of that sensor. SP maps the Challenge vector C[c_1_, c_2_, … , c_m_] and ID of that sensor, constructs PUF Challenge DB, and then sends its own ID and confirmation message to that sensor through TTP, using a secure channel.

As seen in Figure 3b, the gateway sends to TTP along with a requesting message its own ID and IDs of TTP and SP that it wants to register at gateway provisioning stage. Then, TTP selects two large decimal numbers p and q, computes open key n = p * q, and send them back to the gateway so that the gateway and SP can perform zero-knowledge authentication [38]. The gateway selects secret key vector S[s_1_, s_2_, … , s_k_] from 1 < s < n − 1 for zero-knowledge authentication, computes open key vector V[v_1_, v_2_, … , v_k_] = si^2^ mod n (0 < I < k + 1), and sends them back to TTP [39]. TTP that receives the values from the sensor transmits to SP open key vector V[v_1_, v_2_, … , v_k_] and gateway ID. Then SP that receives the values from TTP sends its own ID and a confirmation message to the sensor through TTP and gateway.

#### 3.2.2. Authentication Phase

**Figure 4:**
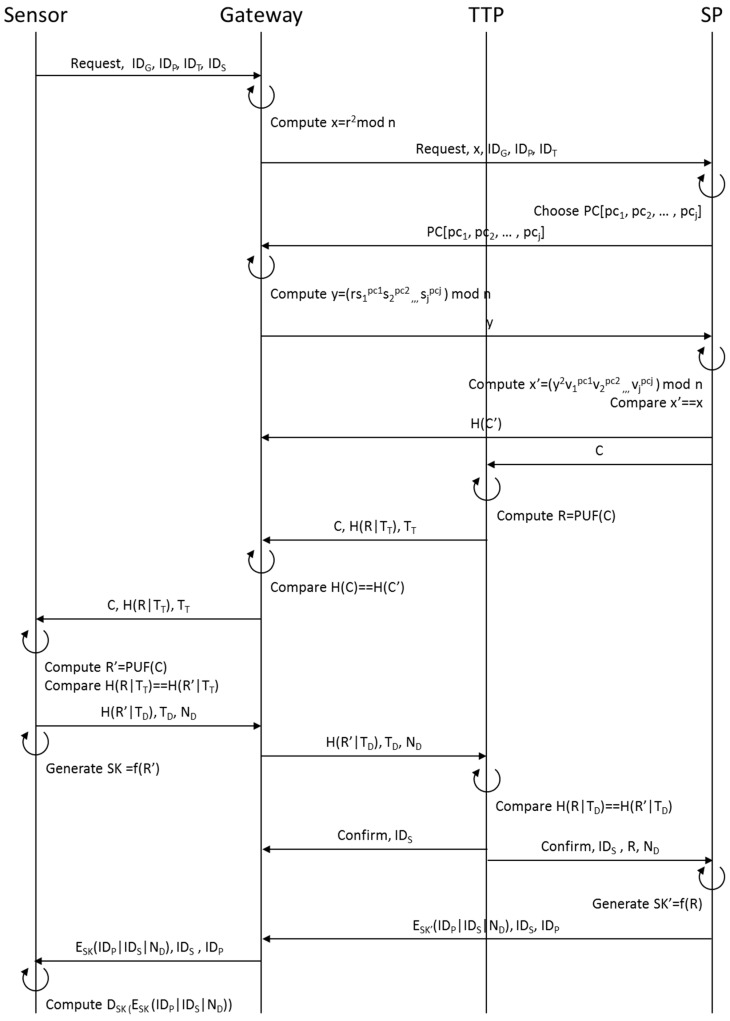
Authentication phase.

Figure 4 shows the authentication phase in which each of the sensors, the gateway, TTP and SP perform mutual authentication on one another and construct a secure channel between the sensor and SP. To construct a secure channel with the SP registered at the provisioning stage, the sensor transmits the IDs of sensor, gateway, TTP and SP to the gateway along with a requesting message. Then, the gateway that receives a request from the sensor computes a Witness value x and x = r^2^ mod n together, which are necessary to perform zero-knowledge proofs with SP and then sends to SP its own ID, IDs of TTP and SP, a requesting message, along with x. SP transmits the Challenge vector PC[pc_1_, pc_2_, … , pc_j_] to the gateway for Zero-knowledge Proofs and the gateway transmits to SP computed Response value y = (rs_1_^pc1^s_2_^pc2^ ,,, s_j_^pcj^) mod n that was computed using the private key of S[s_1_, s_2_, … , s_k_]. Then SP computes x’ = (y^2^v_1_^pc1^v_2_^pc2^, v_j_^pcj^) mod n by using received y value and public key and compares the original x value with the computed x’ value [38,39,40]. If two values are identical, SP grants authentication to the gateway.

After SP authenticates the gateway, it selects C value from PUF Challenge DB matching a relevant sensor to perform PUF Challenge-Response with the ID of the sensor, and then transmits the C value to TTP and H(C’) value (value of hashed C) to the gateway. The TTP that receives the C value from SP computes PUF(C) through the PUF DB to produce R, which is the PUF Response value, hashes R with the current time stamp value T_T,_ and transmits H(R|T_T_) to the gateway along with C and T_T_ to the gateway. The gateway that receives the values from SP hashes C value and checks if the hashed C value is identical with H(C’) value from SP. If the values are identical, the gateway authenticates SP.

After the gateway authenticates SP, the former transmits C, H(R|T_T_) and T_T_ from TTP to the sensor. The sensor that receives the values from the gateway produces R’ = PUF(C) out of PUF embedded in the magnetic device and computes H(R’|T_T_) to confirm whether it is identical with the received H(R’|T_T_). If the values are same, the sensor grants authentication to the gateway [34,35,36].

The sensor that authenticates the gateway hashes the current time stamp value T_D_ and R’, selects random number N_D,_ transmits H(R’|T_D_), T_D_, and N_D_ to TTP via the gateway. Then, it creates a session key SK = f(R’) for SP through the SK generator () [37]. Then, TTP that receives the values from the sensor through the gateway computes H(R|T_D_) with transmitted T_D_ and confirms whether the two H(R|T_D_)s are identical. If they are identical, TTP sends to the gateway the sensor ID and a confirmation message to inform that the sensor is an authentic user and Confirm (message), ID_S_, R, and N_D_ to SP and removes Challenge C and Response R pair from PUF DB. In addition, the gateway and SP that receive the values from TTP authenticate the sensor.

To get authenticated by the sensor and build a secure channel with it, the SP that receives the values from TTP removes the relevant Challenge C from PUF Challenge DB [36]. The SP generates session key SK = f(R) by using R value from TTP, encodes its own ID and the sensor ID with N_D_ into SK, and transmits to the sensor E_SK_’(ID_P_|ID_S_|N_D_), IDS, IDP through the gateway. The sensor that receives the values from SP via the gateway computes the D_SK_(E_SK_(ID_P_|ID_S_|N_D_)) using the session key it created itself. The sensor uses its own creation of the session key SK value to get the D_SK_(E_SK_(ID_P_|ID_S_|N_D_)). If the decrypted values turn out to be ID_P_|ID_S_|N_D_, the sensor authenticates SP and constructs a security channel using the shared SK.

## 4. Security and Performance Analysis

### 4.1. Security Analysis

Table 2 shows whether the proposed as well as existing schemes support multiplatform, is designed to consider the security considering the low resources of the device with small computing and battery power, and whether it protects the user’s privacy. Also, Table 3 shows how much the proposed as well as existing schemes are secure from the various security threats. The proposed protocol provides mutual authentication between sensor and gateway, gateway and SP and sensor and SP. In the proposed protocol, TTP acts as distributed KS that guides the sensors and SPs secure from relay attack, replay attack, eavesdropping, leaked key, and forward security threats, helps them authenticate each other, and share session keys. Besides, when compared with conventional system, the proposed system is more stable and ensures privacy, supporting the low cost of a smart home sensor and the multiplatform environment. 

*Multiplatform*: In this smart home infrastructure, which ensure users’ privacy, smart home sensors with PUF chips, gateway, and service providers have reliable TTP and organically communicate with each other through it. This infrastructure designed TTP to own PUF DB, so multiplatform service providers or sensors can build a secure channel through mutual authentication, without prior information. It designs the gateway to authenticate service providers and sensors with information it receives from TTP and to build a secure channel. As a result, the smart home infrastructure proposed in this paper solves the issue of heterogeneity and prior information exchange in a multiplatform environment.

*Privacy*: Privacy exposure in smart home environment is not an issue of a single individual, but a grave threat to his/her whole family. To tackle problems such as malicious eavesdropping or exposure attributable to user’s recklessness, the proposed smart home infrastructure and protocol proposes building multi-security channels, not simply station-to-station security channels, between smart home sensors and the gateway, between the gateway and the service providers, and between service providers and home sensors, to prevent the leakage of privacy in advance.

*Relay attacks*: A malicious attacker may try to perform a relay attack between a sensor and the gateway or between the gateway and SP, but if the malicious attacker sends an authentication request to the gateway while disguising him/herself as a sensor, the attacker may get the values transmitted from SP, but they are not valid and useful as public values. PUF Response R values necessary for producing a session key are transmitted in a hashed state and can be produced from physically unclonable PUF. Therefore, it can’t build a secure channel with a SP. Even when the malicious attacker disguises him/herself as an SP and then asks a sensor for authentication through the gateway, it cannot create a session key between a sensor and the SP because the attacker has to get PUF Response R values, with which a session key can be produced. Therefore, the session key between a sensor and SP cannot be produced.

*Replay attacks*: A malicious attacker may intercept and re-use for malicious authentication the messages between a sensor and TTP and/or between SPs by conducting replay attacks. In Zero-knowledge proofs which is conducted between the gateway and SP, however, it is impossible for a malicious attacker to perform a replay attack and get access to the message because the gateway computes Witness value x using a random value for authentication and furthermore PUF Challenge-Response pairs exchanged between sensors and SPs are removed every time they are used. In addition, the sensor does not use PUF DB. Although the values used are not removed from the DB, C values cannot be re-used because the Challenge C value is transmitted in a hashed state with a time stamp TT value from the TTP. In addition, when a sensor sends an authentication value to the SP, it sends hashed values with time stamp TT and random value secret ND. Therefore, re-use attacks are impossible.

*Eavesdropping*: A random user may eavesdrop the message exchanges at the mutual authentication stage. Especially, as communication is conducted wirelessly between sensors and the gateway, it can be a big security threat if eavesdropped message contains any confidential information. The clear text exchange in the proposed protocol is composed of public values for zero-knowledge proofs, ID for each authentication entity, non-reused Challenge C, random value, and time stamps, but the Response R value, which is sensitive data required for authentication and creation of session keys, is all hashed before being exchanged.

*Leaked keys*: When the sensor and SP exchange keys for the construction of a secure channel, and their keys are exposed, there can be a lot of problems from the possible exposure of the sensitive information exchanged during the communication. In our proposed protocol, the key is secure because the key required to construct the secure channel between sensor and SP is created through the PUF Response value, that the PUF of the sensor which can get the Response R is physically unclonable, and the server stores the Challenge C value only on PUF DB and receives the R mapped on the Challenge C from TTP only. In addition, when the sensor performs the authentication process with SP through TTP, it has no risk of confidential value exposure even if the TTP server or SP is hacked since it does not expose confidential value to the others by using zero knowledge proofs.

*Mutual authentication*: As the platforms of sensors and SP are uniform in a multiplatform smart home environment, various kinds of issues related to heterogeneity can take place. In the proposed protocol, a gateway is positioned between sensors and SPs as a uniform communication channel and it secures reliability among multiplatform devices by mutual authentication via TTP. The system is designed to provide the mutual authentication between sensor and gateway, gateway and SP, and SP and sensor so that each section of the communication process can be authenticated.

*Forward security*: A malicious attacker may try to take away current session keys between the sensors and SPs and restore past messages. In case the sensors and SPs repeat using a (same) session key at a regular interval, or use parameters that affect past and present in creating a session key, the attacker can restore past session keys, but the proposed protocol does not allow the re-use of PUF Response R values already used in producing sessions, but remove them from the DB every time it is used. Therefore, this system ensures forward security. 

### 4.2. Computing Resource Analysis

Table 4 shows storage the resources calculated under the proposed protocol where there are x sensors, one gateway, Y SPs and one TTP at the provisioning phase and authentication phase. Here, it is assumed that x sensors (x < X) are registered on each SP. The sensor with lowest computing power among the sensors, gateway, TTP and SP does not perform complicated computations (in data volume) and often (in the number of operation), except for m-number of PUF computations in the initial registration process. Even for a gateway with medium computing power, the most complicated computation it performs is limited to Y SP and zero-knowledge proofs. Because SP and TTP have enough computing resources, so they perform more complicated computations than a sensor and a gateway do. However, the most complicated computation for a SP is to carry out zero knowledge proofs with the gateway. To TTP, also, (m + 1) * X PUF is the most complicated computation. Computing load is divided and allocated to gateway, SP, and TTP in the order by computing resources, and it was designed for them to perform mutual authentication and security channel building with minimum computation.

For example, if there are 100 sensors in the smart home, the calculated amount of each sensor is constant as shown in Table 4 regardless of the number of sensors since it authenticates gateway and SP once. The calculated amount at the gateway is proportional to the number of sensors, so it can be O(100^i^) and SP as well according to zero knowledge proofs. It means that SP performs encryption process so as to do more calculations than gateway and TTP has the calculated amount of O(100^i^) in proportion of the number of sensors. Hence, the sensor has the lowest calculated amount followed by TTP, Gateway, and SP. Especially, TTP lowers the burden to perform low amount of calculation while it authenticates with the most sensors.

### 4.3. Storage Resource Analysis

Table 5 shows the storage resources calculated under the proposed protocol where there are x sensors, one gateway, Y SPs and one TTP at the provisioning phase and authentication phase. The sensor of lowest storage capacity has its own ID and the IDs of gateway, TTP and SP to which they belong, and stores five additional parameters for the authentication and secure channels. The gateway owns its own ID, X sensor IDs and Y server IDs as well as two parameters for authentication. SP has its own ID and one respective ID for gateway and TTP, and x sensors’ IDs. In addition, it possesses mx Challenge C values and x time stamp parameters to perform the authentication with sensors and build secure channels. TTP has its own ID and IDs for x sensors, Y SPs and a gateway, and mX challenge-response pairs to authenticate x sensors. By capacity, storage resources were allocated to sensor, gateway, SP, and TTP in this order and minimum storage capacity was allocated to a sensor lacking storage resources the most. 

If 100 sensors should save 128 bytes passwords in each entry to authenticate to TTP, SP, or gateway, SP, gateway, and TTP would require the storage space with 128 bytes * 100 units = 12,800 bytes by each. However, it does not have to store the passwords in TTP or SP by using zero knowledge proofs, so it can save storage space.

## 5. Conclusions

This paper deals with increasing heterogeneity in multiplatform scenarios and security threats as the smart home market is exponentially growing. It proposes a scheme by which mutual authentication and secure channels can be efficiently constructed with minimum resources. The proposed TTP-based infrastructure can not only provide reliability to multiplatform entities participating in remote communication requiring mutual authentication, but also help sensors of low computing capacity and SPs that have to process data from an ever-increasing number of sensors while minimizing resource consumption in carrying out authentication with the sensors. Using PUFs, it also minimizes the consumption of computing resources by enabling smart home sensors to process authentication and producing session keys without additional hardware. In this paper, it was confirmed through security assessment that the proposed system is secure from common threats. Also, the proposed scheme was analyzed and tested for its computing and storage resources needed by communication entity. At present, the smart home technology remains at the level of “connected home”. However, if it is openly applied to markets such as mobile app stores, multiplatform environments for smart home will become more diversified than now, shifting away from one-way service provision as it is now. It is hoped that the scheme proposed in the paper will help build secure communication with fewer resources in future multiplatform smart home environment. 

## Figures and Tables

**Table 1:** Proposed Protocol Parameters.

Notation	Meaning
Sensor	Smart home Sensor Device
Gateway	Smart home Gateway
TTP	Trusted Third Party
SP	Service Provider
ID_S_, ID_G_, ID_T_, ID_P_	Sensor, Gateway, TTP and SP ID
PUF	Physical unclonable function
C[c_1_, c_2_, … , c_m_]	Challenge vector value for PUF
R[r_1_, r_2_, … , r_m_]	Response vector value for PUF
S[s_1_, s_2_, … , s_k_]	Gateway private key vector for Zero-knowledge Proofs
V[v_1_, v_2_, … , v_k_]	Gateway public key vector for Zero-knowledge Proofs
PC[pc_1_, pc_2_, … , pc_j_]	Challenge vector for zero-knowledge Proofs
n	Public key for Zero-knowledge Proofs
r	Random value for Zero-knowledge Proofs
x	Witness value for zero-knowledge Proofs
y	Response value for zero-knowledge Proofs
T	Timestamp
N	Nonce
SK	Session key between Sensor and SP
H()	Hash function
f()	SK generator
D()	Decryption
E()	Encryption

**Table 2:** Comparative security analysis between smart home protocols.

	Jinsung et al. [30]	Alessandro et al. [31]	Khusvinder et al. [32]	Vijay et al. [33]	Proposed Scheme
Multiplatform	X	O	X	X	O
Low Resource	O	O	Δ	X	O
Security	X	X	O	O	O
Privacy	X	X	Δ	O	O

O: Support; Δ: not fully support; X: Not support.

**Table 3:** Comparative performance analysis between sensor protocols.

	ELK [22]	LKH [23]	CoGKTK [24]	sGIM [25]	Proposed Scheme
Distributed KS	Not-support	Not-support	Support	Support	Support
Forward Security	X	X	O	X	O
Mutual Authentication	X	X	O	X	O
Relay Attack	X	X	X	X	O
Replay Attack	X	X	O	O	O

O: Secure; X: Vulnerable.

**Table 4:** Comparative computing resource analysis between communication objects.

	Sensor	Gateway	SP	TTP
PUF	m+1	-	-	(m + 1) * X
Hash	1	1	-	2X
Encryption	1	-	x	-
Decryption	1	-	x	-
f()	1	-	x	-
Nonce generation	1	k	-	(m + 2) * X
Zero-knowledge Proofs computations	-	O(n^i^)	O(n^j^)	-

**Table 5:** Comparative storage resource analysis between communication objects.

	Sensor	Gateway	SP	TTP
ID	4	2 + Y + X	3 + x	2 + Y + X
Challenge C	1	1	mx	mX
Response R	1	-	-	mX
SK	1	-	x	-
Timestamp	1	-	-	X
Nonce	1	1	-	-

## Abbreviations

IoT	Internet of Things
ZiSAS	ZigBee-based intelligent self-adjusting sensor
WSNs	Wireless Sensor Networks
RHS	Remote Home Server
SDN	Software Defined Networking
SMP	Security Management Provider
PUFs	Physical Unclonable Functions
TTP	Trusted Third Party
SP	Service Provider
